# The proportion and phenotypic changes of CD4^+^CD25^−^Foxp3^+^ T cells in patients with untreated rheumatoid arthritis

**DOI:** 10.1186/s12865-022-00517-0

**Published:** 2022-09-06

**Authors:** Bomiao Ju, Li Zhu, Jing Wang, Jian Zheng, Zhiming Hao, Jing Luo, Jing Zhang, Nan Hu, Qi An, Xiuyuan Feng, Yongwei Huo, Lan He

**Affiliations:** 1 Department of Rheumatology and Immunology, The First Affiliated Hospital of Xi’an Jiaotong University, Xi’an, 710061 Shaanxi China; 2 Department of Human Anatomy and Histology and Embryology, School of Basic Medical Sciences, Xi’an Jiaotong University Health Science Center, Xi’an, 710061 Shaanxi China

**Keywords:** Rheumatoid arthritis, CD4^+^CD25^−^Foxp3^+^ T cells, CD4^+^CD25^+^Foxp3^+^ T cells, Disease activity, Phenotypic

## Abstract

**Objective:**

CD4^+^CD25^+^Foxp3^+^ regulatory T (Treg) cell-mediated immunosuppression is an essential mechanism of rheumatoid arthritis (RA). However, little is known regarding the specific role of CD4^+^CD25^−^Foxp3^+^ Treg cells in RA. This study aimed to investigate the frequency of circulating CD4^+^CD25^−^Foxp3^+^ Treg cells and their role in RA.

**Methods:**

Sixty-one untreated RA patients and 40 healthy controls (HCs) were enrolled in this study. The proportion of CD4^+^CD25^−^Foxp3^+^ T cells and CD4^+^CD25^+^Foxp3^+^ Tregs; the levels of CTLA4, GITR, Helios, and ICOS; and the production of IL-17A, IFN-γ, and IL-10 were assessed by flow cytometry. The correlation of CD4^+^CD25^–^Foxp3^+^ T cells and CD4^+^CD25^+^Foxp3^+^ Tregs with the clinical indicators was conducted by Spearman correlation analysis.

**Results:**

The proportion of CD4^+^CD25^–^Foxp3^+^ T cells was elevated in RA and positively correlated with disease activity. CD4^+^CD25^–^Foxp3^+^ T cells expressed less Helios and produced more IFN-γ than conventional Tregs in RA. Additionally, the proportion of CD4^+^CD25^–^Foxp3^+^ T cells was positively correlated with DAS28 score, IgG titer, and anti-CCP titer.

**Conclusions:**

These data indicate that CD4^+^CD25^−^Foxp3^+^ T cells in RA exhibit several different functional properties from conventional Tregs and are correlated with RA disease activity.

## Highlights

- The proportion of CD4^+^CD25^−^Foxp3^+^ T cells was elevated in untreated RA.
- CD4^+^CD25^−^Foxp3^+^ T cells expressed decreased Helios compared with Treg cells in both RA patients and HCs.
- CD4^+^CD25^−^Foxp3^+^ T cells produced increased IFN-γ compared with Tregs in RA patients.
- Elevated CD4^+^CD25^−^Foxp3^+^ T cells were positively correlated with DAS28 score, anti-IgG titer and anti-CCP titer.

## Introduction

Rheumatoid arthritis (RA) is considered a chronic systemic autoimmune disease with excessive activation of collagen-specific T helper cells and elevated levels of autoantibodies in the serum [1]. CD4^+^CD25^+^Foxp3^+^ regulatory T cells (Tregs) can suppress the activity of autoreactive T cells that have escaped from the thymus and are involved in the pathogenesis of RA. Recently, a subgroup of CD4^+^FoxP3^+^ T cells negative for CD25 has been detected in multiple immune regulatory diseases, such as systemic lupus erythematosus (SLE) [2], HIV and *Mycobacterium tuberculosis* infection [3], and non-Hodgkin lymphoma (NHL). Bonelli M et al. demonstrated CD4^+^CD25^–^Foxp3^+^ T cells and Tregs with a similar phenotype [4]. However, Yang HX et al. proved that CD4^+^CD25^–^Foxp3^+^ T cells had suppressive activity similar to that of effector T cells and secreted IFN-γ, IL-4, IL-2, and IL-17A, although less than that of effector T cells in SLE patients [5]. An increased proportion of CD4^+^CD25^–^Foxp3^+^ T cells has also been reported in RA [6]. However, the functional features and clinical significance of CD4^+^CD25^−^Foxp3^+^ T cells remain unclear.

Therefore, the present study investigated the functional features of CD4^+^CD25^–^Foxp3^+^ T cells and their correlation with clinical indicators in RA patients. PBMCs were isolated from 61 untreated RA patients and 40 HCs to evaluate the proportion of CD4^+^CD25^−^Foxp3^+^ T cells in RA patients. Spearman correlation analysis was conducted to investigate the correlation between CD4^+^CD25^–^Foxp3^+^ T cells and clinical indicators. Moreover, the expression of the Treg-associated functional molecules CTLA-4 (cytotoxic T-lymphocyte associated protein 4), GITR (glucocorticoid-induced tumor necrosis factor receptor), ICOS (inducible costimulator) and Helios and the production of cytokines (IFN-γ, IL-17A, and IL-10) were analyzed to investigate the functional features of CD4^+^CD25^–^Foxp3^+^ T cells.

## Methods

### Patients

Sixty-one untreated RA and 40 HCs of comparable sex and age were recruited between October 2017 and December 2020 at the Department of Rheumatology and Immunology, the First Affiliated Hospital of Xi'an Jiaotong University. The inclusion criteria were as follows: (1) met the American College of Rheumatology/European League Against Rheumatism 2010 diagnostic criteria [7]; (2) disease-modifying anti-rheumatic drug (DMARD)-naïve patients. DMARDs included basic commonly accepted treatments and glucocorticoids. The exclusion criteria were as follows: (1) the use of any DMARD therapy; (2) pregnancy; and (3) an acute infection or any severe infection.

The disease activity index of rheumatoid arthritis was assessed by the validity of the 28-joint disease activity score using erythrocyte sedimentation rate (DAS28-ESR). RA patients were divided into the inactive RA group (DAS28 < 2.6) and active RA group (DAS28 ≥ 2.6) according to the degree of disease activity [8]. The basic characteristics and the levels of erythrocyte sedimentation rate (ESR), C-reactive protein (CRP), rheumatoid factors (RFs), and anti-citrullinated protein antibody (anti-CCPs) were obtained on the day of sample collection. The clinical characteristics of the RA patients and HCs are shown in Table 1. Written consent for the participation of these subjects was obtained. The study protocol was approved by the ethics committee of the First Affiliated Hospital of Xi’an Jiao Tong University (Ethics number KYLLSL-2018-207).

**Table 1:** Characteristics of patients with RA patients and Healthy controls (HCs)

Clinical variables	RA patients (n = 61)	HCs (n = 40)
Sex, female/male (n)	51/10	37/3
Age, years mean ± SEM	43.15 ± 15.46	44.68 ± 13.39
Disease duration, months	10 (4–36)	
ESR, mm/h	27 (16–41.5)	
CRP, mg/l	6.95 (3.20–17.4)	
DAS28-ESR	4.39 (2.83–5.58)	
DAS28-CRP	3.64 (3.01–4.92)	
Tender joint count	6 (3.5–15)	
Swollen joint count	7.5 (2.75–17.5)	
Anti-CCP, IU/ml	21.9 (0.63–266.9)	
Anti-CCP, positivity	50/61 (82.0%)	
RF, IU/ml	27.5 (12.0–160.8)	
RF, positivity	43/61 (70.5%)	

All the data were demonstrated as median [IQR, 25th–75th percentile]

RA, rheumatoid arthritis; DAS28-ESR, Disease Activity Score in 28 joints using the erythrocyte sedimentation rate; CRP, C-reactive protein; RF, rheumatoid factor; anti-CCP, anti-cyclic citrullinated peptide

### Flow cytometric analysis of the proportion of CD4^+^CD25^***−***^Foxp3^+^ T cells and CD4^+^CD25^+^Foxp3^+^ Treg cells

PBMCs were isolated from venous blood (2 ml) using Ficoll-Paque™ PREMIUM (GE Healthcare Life Sciences) density-gradient centrifugation. FITC-conjugated CD4 (BD Pharmingen™, cat: 555346), PE-conjugated CD25 (BD Pharmingen™, cat: 555432), and PerCP-CyTM-conjugated CD127 (BD Pharmingen™, cat: 560551) were used for surface staining to evaluate the proportion of CD4^+^CD25^−^Foxp3^+^ T cells and CD4^+^CD25^+^Foxp3^+^ Treg cells. The cells were then fixed and permeabilized using 1 × Foxp3 Perm buffer (BD Pharmingen™) for intracellular Alexa Fluor 647-conjugated FoxP3 staining (BD Pharmingen™, cat: 560045). The proportions of CD4^+^CD25^−^Foxp3^+^ T cells and CD4^+^CD25^+^Foxp3^+^ Treg cells were acquired on a BD FACS Canto II and analyzed using FlowJo software (version 7.6.1; Tree Star).

### Phenotypic and cytokine expression analysis of Treg cells

PC5.5-conjugated CD4 (BD Pharmingen™; cat: 560650), PC7-conjugated CD25 (BD Pharmingen™; cat: 557741), PB450-conjugated ICOS (BD Pharmingen™; cat: 562901) or PB450-conjugated GITR (BD Pharmingen™; cat: 747658) were used for surface staining. Consequently, the cells were fixed and permeabilized. PE-conjugated Foxp3 (BD Pharmingen™; cat: 560650), APC-conjugated CTLA4 (BD Pharmingen™; cat: 555855) or APC-conjugated Helios (BD Pharmingen™; cat: 560046) were used for intracellular staining.

To analyze the production of cytokines, PBMCs (2 × 10^6^ cells/ml) were incubated in RPMI 1640 culture medium containing 10% fetal bovine serum (Sigma; cat no. 030M3399) with 50 ng/ml phorbol myristate acetate (PMA) (Sigma; cat: P1585-1MG) and 1 µg/ml ionomycin (Sigma; cat no. I0634-1MG) in the presence of 0.7 µl/mL GolgiStop (BD Biosciences; cat: 554724) and 1 µl/mL Golgi Plug (BD Biosciences; cat: 555029) for 6 h according to the manufacturer’s instructions. Surface staining for CD4 and CD25 and intracellular staining for Foxp3, PB450-conjugated IL-17A (BD Pharmingen™; cat: 562933), FITC-conjugated IFN-γ (BD Pharmingen™; cat: 554700), or APC–conjugated IL-10 (BD Pharmingen™; cat: 554707) were the same as described above.

### Statistical analysis

Statistical analysis was performed using SPSS Statistics 22 software and GraphPad Prism 5.0 software (GraphPad Software, San Diego, CA). The Mann–Whitney U test and the Kruskal–Wallis test were used to evaluate differences between groups. Correlations were analyzed using *Spearman* correlation analysis. *P* values less than 0.05 were considered to be significant.

## Results

### Elevated proportions of CD4^+^CD25^***−***^Foxp3^+^ T cells and CD4^+^CD25^+^Foxp3^+^ Treg cells in RA patients

**Fig. 1:**
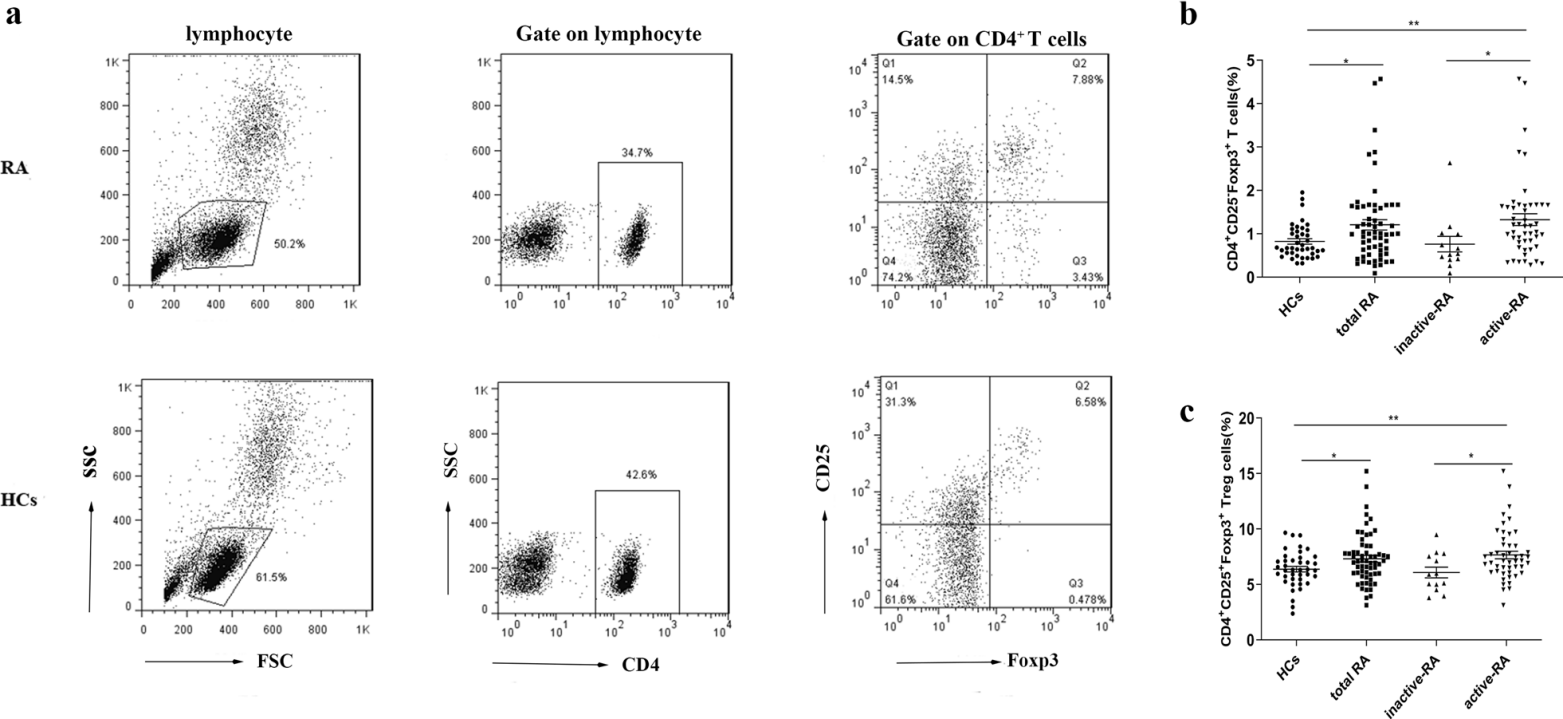
Elevated proportions of CD4+CD25-Foxp3+ T cells and CD4^+^CD25^+^Foxp3^+^ Treg cells in RA: **a** representative flow cytometry plots. **b** The proportion of CD4^+^CD25^−^Foxp3^+^ T cells in the PB of HCs, total untreated RA patients, inactive RA patients, and active RA patients. **c** The proportion of CD4^+^CD25^+^Foxp3^+^ Treg cells in the PB of HCs, total untreated RA patients, inactive RA patients and active RA patients. *p ≤ 0.05, **p ≤ 0.01, ***p ≤ 0.001. HCs, healthy controls; RA, rheumatoid arthritis.

The median DAS28-ESR of the untreated RA patients was 4.39 (2.83–5.58). In our group, 48 patients (78.7%) were assessed as having high disease activity, and 13 patients (21.3%) were assessed as having remission. We analyzed Foxp3 in CD4^+^ lymphocytes subdivided according to the intensity of CD25 expression into CD4^+^CD25^+^Foxp3^+^ Treg cells and CD4^+^CD25^−^Foxp3^+^ T cells (Fig. 1a). The proportions of CD4^+^CD25^−^Foxp3^+^ T cells [1.00 (0.58–1.62) % vs. 0.67 (0.55–1.07) %; *P* < 0.05] and CD4^+^CD25^+^Foxp3^+^ Treg cells [7.07 (5.92–8.18) % vs. 6.31 (5.47–7.34) %; *P* < 0.05] were both significantly higher in the total RA patients than in the HCs. The proportions of CD4^+^CD25^+^Foxp3^+^ Treg cells and CD4^+^CD25^−^Foxp3^+^ T cells were further investigated in active RA patients and inactive RA patients. The proportion of CD4^+^CD25^+^Foxp3^+^ Treg cells [7.14 (6.12–8.55) % vs. 5.89 (4.75–7.65) %; *P* < 0.05] and CD4^+^CD25^−^Foxp3^+^ T cells [1.11 (0.73–1.66) % vs. 0.54 (0.41 − 1.00) %; *P* < 0.01] was higher in the active RA group than in the inactive RA group, and no difference was observed between the inactive RA group and HCs (Fig. 1b, c).

### Phenotypic analysis of CD4^+^CD25^***−***^Foxp3 + T cells and CD4^+^CD25^+^Foxp3^+^ Treg cells

To determine whether the phenotype of CD4^+^CD25^−^Foxp3^+^ T cells resembled that of CD4^+^CD25^+^Foxp3^+^ Treg cells or CD4^+^CD25^+^Foxp3^−^ effector T cells, we investigated molecules associated with regulation of Treg suppressive function, including CTLA-4, GITR, Helios, and ICOS, in untreated RA patients and HCs. The proportions of distinct phenotype makers were presented in Table 2. All the data were demonstrated as median [IQR, 25th-75th percentile]. The expression of Helios [58.52 (50.70–63.42) % vs. 76.18 (72.28–80.39) %; *p* < 0.001] on CD4^+^CD25^−^Foxp3^+^ T cells was significantly lower than that on CD4^+^CD25^+^Foxp3^+^ Treg cells in the RA group. However, no significant differences in the expression levels of CTLA4 [36.36 (28.29–42.95) % vs. 40.40 (27.40–54.00) %; *p* > 0.05], GITR [51.52 (32.35–61.85) % vs. 35.92 (27.18–61.66) %; *p* > 0.05] or ICOS [40.74 (26.86–47.78) % vs. 44.96 (35.15–51.90) %; *p* > 0.05] were identified between CD4^+^CD25^−^Foxp3^+^ T cells and CD4^+^CD25^+^Foxp3^+^ Treg cells in RA patients. The expression levels of CTLA4 [36.36 (28.29–42.95) % vs. 10.61 (5.93–24.91) %; *p* < 0.01], Helios [58.52 (50.70–63.42) % vs. 10.18 (5.04–20.56) %; *p* < 0.001] and ICOS [41.44 (30.73–53.78) % vs. 26.14 (15.78–30.67) %; *p* < 0.01] were upregulated on CD4^+^CD25^−^Foxp3^+^ T cells compared with CD4^+^CD25^+^Foxp3^−^ effector T cells. This finding indicated that CD4^+^CD25^−^Foxp3^+^ T cells share several properties with CD4^+^CD25^+^Foxp3^+^ conventional Tregs in the RA group.

**Table 2:** Phenotypic analysis of CD4^+^CD25^−^Foxp3 + T cells and CD4^+^CD25^+^Foxp3^+^ Treg cells

Phenotypic biomarkers	HCs	RA	*p* value (HCs vs RA)
CTLA4%/CD4^+^CD25^−^Foxp3^+^ T cells	56.69 (46.61–67.39)*†	36.36 (28.29–42.95)†	0.002
CTLA4%/CD4^+^CD25^+^Foxp3^+^ Treg cells	70.50 (60.25–74.35)	40.40 (27.40–54.00)	< 0.001
CTLA4%/CD4^+^CD25^+^Foxp3^−^ effector T cells	3.12 (2.49–12.83)	10.61 (5.93–24.91)	0.156
GITR% /CD4^+^CD25^−^Foxp3^+^ T cells	61.40 (56.84–70.87)*	51.52 (32.35–61.85)	0.048
GITR% /CD4^+^CD25^+^Foxp3^+^ Treg cells	75.03 (64.58–76.91)	35.92 (27.18–61.66)	0.001
GITR% /CD4^+^CD25^+^Foxp3^−^ effector T cells	52.08(50.73–65.55)	53.18(27.60–66.13)	0.615
ICOS%/CD4^+^CD25^−^Foxp3^+^ T cells	44.96 (35.15–51.90)†	40.74 (26.86–47.78)†	0.763
ICOS%/CD4^+^CD25^+^Foxp3^+^ Treg cells	40.74(26.86–47.78)	33.94 (30.60–51.11)	0.971
ICOS%/CD4^+^CD25^+^Foxp3^−^ effector T cells	20.00 (13.60–30.17)	26.14 (15.78–30.67)	0.280
Helios%/CD4^+^CD25^−^Foxp3^+^ T cells	70.18 (53.33–75.60)†	58.52 (50.70–63.42) *†	0.220
Helios%/CD4^+^CD25^+^Foxp3^+^ Treg cells	73.35(58.02–85.93)	76.18 (72.28–80.39)	0.790
Helios%/CD4^+^CD25^+^Foxp3^−^ effector T cells	31.11 (3.99–42.60)	10.18 (5.04–20.56)	0.097

All the data were demonstrated as median [IQR, 25th-75th percentile]

HCs, healthy controls; RA, rheumatoid arthritis; CTLA4, cytotoxic T-lymphocyte associated protein 4; GITR, glucocorticoid-induced tumor necrosis factor receptor; ICOS, inducible T-cell costimulator; Helios, IKAROS family zinc finger 2

*Indicates significance (*p* < 0.05) compared with CD4^+^CD25^+^Foxp3^+^ Treg cells in RA group or HCs

^†^Indicates significance (*p* < 0.05) compared with CD4^+^CD25^+^Foxp3^−^ effector T cells in RA group or HCs

In addition, the expression levels of CTLA-4 [56.69 (46.61–67.39) % vs. 70.50 (60.25–74.35) %; *p* < 0.05] and GITR [61.40 (56.84–70.87) % vs. 75.03 (64.58–76.91) %; *p* < 0.05] were significantly decreased in CD4^+^CD25^−^Foxp3^+^ T cells compared with CD4^+^CD25^+^Foxp3^+^ T cells in HCs. Moreover, the expression levels of CTLA-4 [56.69 (46.61–67.39) % vs. 3.12 (2.49–12.83) %; *p* < 0.001], Helios [70.18 (53.33–75.60) % vs. 31.11 (3.99–42.60) %; *p* < 0.01] and ICOS [44.96 (35.15–51.90) % vs. 20.00 (13.60–30.17) %; *p* < 0.001] were significantly higher in CD4^+^CD25^−^Foxp3^+^ T cells compared with CD4^+^CD25^+^Foxp3^−^ effector T cells in HCs.

We further evaluated these molecules in the RA group compared with HCs. The expression levels of CTLA4 [36.36 (28.29–42.95) % vs. 56.69 (46.61–67.39) %; *p* < 0.01] and GITR [51.52 (32.35–61.85) % vs. 61.40 (56.84–70.87) %; *p* < 0.05] in CD4^+^CD25^−^Foxp3^+^ T cells were lower in RA patients than in HCs. Similarly, the expression levels of CTLA4 [40.40 (27.40–54.00) % vs. 70.50 (60.25–74.35) %; *p* < 0.01] and GITR [35.92 (27.18–61.66) % vs. 75.03 (64.58–76.91) %; *p* < 0.01] in CD4^+^CD25^+^Foxp3^+^ Tregs were significantly decreased in RA patients compared with HCs.

### Cytokines analysis of CD4^+^CD25^***−***^Foxp3^+^ T cells and CD4^+^CD25^+^Foxp3^+^ Treg cells

Furthermore, the cytokine profiles of IL-10, IL-17A, and IFN-γ were determined in CD4^+^CD25^+^Foxp3^+^ Treg cells, CD4^+^CD25^−^Foxp3^+^ T cells, and CD4^+^CD25^+^Foxp3^−^ effector T cells (Table 3). The production of IFN-γ was higher in CD4^+^CD25^−^Foxp3^+^ T cells than in CD4^+^CD25^+^Foxp3^+^ Treg cells and CD4^+^CD25^+^Foxp3^−^ effector T cells in both the RA group [10.34 (5.96–16.54) % vs. 3.72 (2.73–5.73) % vs. 6.83 (3.44–12.66) %; *p* < 0.05] and HCs [9.87 (4.61–11.20) % vs. 2.96 (1.57–4.28) % vs. 3.62 (3.54–4.32) %; *p* < 0.05]. In the HC group, the production of IL-17A [3.20 (1.85–5.51) % vs. 14.98 (8.58–19.07) %; *p* < 0.05] in CD4^+^CD25^−^Foxp3^+^ T cells was significantly lower than that in CD4^+^CD25^+^Foxp3 effector T cells. No significant difference in the production of IL-10 in CD4^+^CD25^−^Foxp3^+^ T cells, CD4^+^CD25^+^Foxp3^+^ Treg cells or CD4^+^CD25^+^Foxp3^−^ effector T cells was observed in either RA or HCs.

**Table 3:** Cytokines analysis of CD4^+^CD25^−^Foxp3^+^ T cells and CD4^+^CD25^+^Foxp3^+^ Treg cells

Cytokines	HCs	RA	*p* value (HCs vs RA)
IL-10%/CD4^+^CD25^−^Foxp3^+^ T cells	28.33 (15.20–36.53)	29.43 (18.72–43.84)	0.651
IL-10%/CD4^+^CD25^+^Foxp3^+^ Treg cells	21.69 (16.48–28.50)	20.36 (13.34–33.86)	0.778
IL-10%/CD4^+^CD25^+^Foxp3^−^ effector T cells	21.25 (15.22–29.93)	15.24 (11.20–25.51)	0.254
IL-17A% /CD4^+^CD25^−^Foxp3^+^ T cells	3.20 (1.85–5.51)†	6.19 (3.94–8.12)	0.048
IL-17A%/CD4^+^CD25^+^Foxp3^+^ Treg cells	2.62 (1.58–5.36)	6.18 (5.03–13.34)	0.009
IL-17A% /CD4^+^CD25^+^Foxp3^−^ effector T cells	14.98 (8.58–19.07)	7.76 (5.27–20.07)	0.334
IFN-γ %/CD4^+^CD25^−^Foxp3^+^ T cells	9.87 (4.61–11.20)*†	10.34 (5.96–16.54)*†	0.651
IFN-γ%/CD4^+^CD25^+^Foxp3^+^ Treg cells	2.96 (1.57–4.28)	3.72 (2.73–5.73)	0.395
IFN-γ%/CD4^+^CD25^+^Foxp3^−^ effector T cells	3.62 (3.54–4.32)	6.83 (3.44–12.66)	0.279

All the data were demonstrated as median [IQR, 25th-75th percentile]

HCs, healthy controls; RA, rheumatoid arthritis; IL-10, interleukin 10; IL17-A, interleukin 17A; IFN-γ, interferon gamma

*Indicates significance (*p* < 0.05) compared with CD4^+^CD25 ^+^Foxp3^+^ Treg cells in RA group or HCs

^†^Indicates significance (*p* < 0.05) compared with CD4^+^CD25^+^Foxp3^−^ effector T cells in RA group or HCs

We further compared the production of cytokines in CD4^+^CD25^−^Foxp3^+^ T cells and CD4^+^CD25^+^Foxp3^+^ Tregs in the RA group compared with HCs. The CD4^+^CD25^+^Foxp3^+^ Treg cells [6.18 (5.03–13.34) % vs. 2.62 (1.58–5.36) %; *p* < 0.01] and CD4^+^CD25^−^Foxp3^+^ T cells [6.19 (3.94–8.12) % vs. 3.20 (1.85–5.51) %; *p* < 0.01] produced more IL-17A in the RA group than in the HC group.

### The relationship of CD4^+^CD25^***−***^Foxp3^+^ T cells and CD4^+^CD25^+^Foxp3^+^ Treg cells with RA clinical indicators

**Fig. 2:**
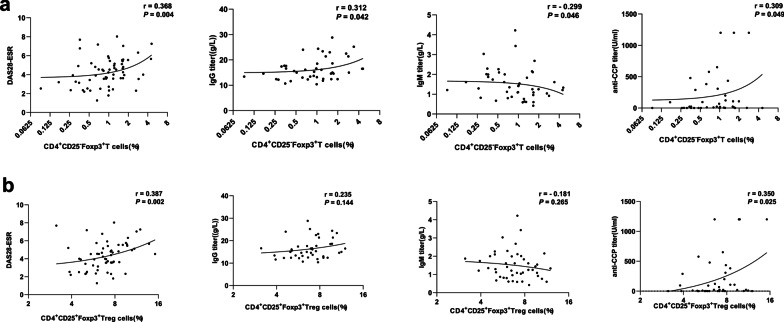
The correlation between the proportions of CD4^+^CD25^−^Foxp3^+^ T cells and CD4^+^CD25^+^Foxp3^+^ Treg cells and clinical variables. **a**, **b** The Spearman rank test was used to analyse the correlation between the CD4^+^CD25^−^Foxp3^+^ T cells or CD4^+^CD25^+^Foxp3^+^ Treg cells and DAS28-ESR, IgG titer, IgM titer, and anti-CCP titer.

The relationship between CD4^+^CD25^−^Foxp3^+^ T cells and CD4^+^CD25^+^Foxp3^+^ Treg cells and RA clinical indicators was further studied in these RA patients. As shown in Fig. 2a, the proportion of CD4^+^CD25^−^Foxp3^+^ T cells was positively correlated with the DAS28-ESR (r = 0.368; *P* = 0.004), serum IgG titer (r = 0.312; *P* = 0.042) and anti-CCP titer (r = 0.309; *P* = 0.049) and negatively correlated with IgM titer (*r* = − 0.299; *P* = 0.046). The proportion of CD4^+^CD25^+^Foxp3^+^ Treg cells was positively correlated with DAS28-ESR (r = 0.387; *P* = 0.002) and anti-CCP titer (r = 0.350; *P* = 0.025) (Fig. 2b). Our data also show that the proportion of CD4^+^CD25^−^Foxp3^+^ T cells was positively correlated with the swollen joint counts (r = 0.257; *P* = 0.046), patient reported general health (VAS) scores (r = 0.268; *P* = 0.037) and DAS28-CRP (r = 0.299; *P* = 0.019). No correlation found between tender joint counts and CD4^+^CD25^−^Foxp3^+^ T cells (r = 0.215; *P* = 0.096).

## Discussion

This study demonstrated that the proportion of CD4^+^CD25^−^Foxp3^+^ T cells was elevated in untreated RA. The CD4^+^CD25^−^Foxp3^+^ T cells expressed decreased Helios compared with Treg cells in both RA patients and HCs. CD4^+^CD25^−^Foxp3^+^ T cells produced higher levels of IFN-γ than Tregs in RA patients. Elevated CD4^+^CD25^−^Foxp3^+^ T cells were positively correlated with DAS28, anti-IgG titer, and anti-CCP titer. These results indicate that CD4^+^CD25^−^Foxp3^+^ T cells exhibit several different functional properties from conventional Tregs.

The elevated proportions of CD4^+^CD25^−^Foxp3^+^ T cells detected in our study are consistent with Banesa de Paz. et al., who reported the enrichment of CD4^+^CD25^−^Foxp3^+^ T cells in 147 RA patients [9]. Our study also observed elevated proportions of CD4^+^CD25^+^Foxp3^+^ Treg cells in untreated RA patients compared with HCs. These results were consistent with several studies described previously [10–12]. However, Cao D, Sempere-Ortells JM, Samson Mand Kosmaczewska A, et al. reported that the proportion of Treg cells was lower in RA patients [13, 14]. The heterogeneity of RA disease, the markers identifying Treg cells, and treatment agents could be the basis for this discrepancy. This study analyzed untreated RA patients to avoid drug interference and used CD4 + CD25^high^ and Foxp3 expression as markers of Treg cells. Furthermore, our study indicated that the proportions of CD4^+^CD25^+^Foxp3^+^ Treg cells and CD4^+^CD25^−^Foxp3^+^ T cells were increased in patients with active RA compared with those with inactive RA. In addition, the proportions of CD4^+^CD25^+^Foxp3^+^ Treg cells and CD4^+^CD25^−^Foxp3^+^ T cells were both associated with DAS28-ESR. Therefore, we speculated that the increased proportion of CD4^+^CD25^+^Foxp3^+^ Treg cells and CD4^+^CD25^−^Foxp3^+^ T cells might be due to feedback of the hyperinflammatory state in RA.

Inhibitory-related molecules are pivotal for the maintenance of Treg cell homeostasis and suppressive function [15]. Therefore, inhibitory-related molecules (CTLA-4, GITR, ICOS, and Helios) were analyzed on CD4^+^CD25^−^Foxp3^+^ T cells and Treg cells. CTLA-4 is constitutively expressed on Treg cells and can inhibit the expression of the costimulatory molecules CD80 and CD86 on antigen-presenting cells to suppress the activation of effector T cells [16]. GITR participates in the intransient inhibition of Treg activity and stimulation of Treg proliferation [17]. Helios, a member of the Ikaros gene transcription factor family, is expressed in a subset of Foxp3^+^ Tregs, which ensures Treg cells have a suppressive and anergic phenotype in the face of intense inflammatory responses. The defective expression of Helios could lead to the conversion of Treg cells into an effector T-cell phenotype that produces proinflammatory cytokines [18]. ICOS is a member of the CD28 family of costimulatory molecules and has a key role in controlling the effector functions of Treg cells [19]. Cytokine production is another important way Tregs perform suppressive functions. Treg cells are conventionally associated with the production of classical anti-inflammatory cytokines, such as IL-10, IL-35, and TGF-β. In contrast, Treg cells can also produce effector cytokines, including IFN-γ and IL-17A, under inflammatory conditions [20]. Therefore, we explored the expression of Treg-associated molecules (CTLA-4, GITR, ICOS and Helios) and the cytokine (IFN-γ, IL-17A, and IL-10) synthesis capacity in CD4^+^CD25^–^Foxp3^+^ T cells to investigate the functional features of CD4^+^CD25^–^Foxp3^+^ T cells. Our results showed that CD4^+^CD25^−^Foxp3^+^ T cells expressed less Helios and more IFN-γ than CD4^+^CD25^+^Foxp3^+^ Treg cells in RA patients. However, the expression of CTLA4, GITR, and ICOS and the production of IL-17A and IL-10 by CD4^+^CD25^−^Foxp3^+^ T cells were similar to those by CD4^+^CD25^+^Foxp3^+^ Treg cells in RA patients. These findings may indicate that CD4^+^CD25^−^Foxp3^+^ T cells have certain characteristics of Treg cells in RA patients. In addition, our results showed that the expression levels of CTLA4, Helios, and ICOS on CD4^+^CD25^−^Foxp3^+^ T cells were higher than those on CD4^+^CD25^+^Foxp3^−^effector T cells. These results raise the possibility that CD4^+^CD25^−^Foxp3^+^ T cells in RA may serve as intermediates between Treg cells and effector T cells, which combine their features. Previous studies in SLE have reached different conclusions. One study suggested that CD4^+^CD25^−^Foxp3^+^ T cells may have lower inhibitory function than Treg cells [21]. Another study suggested that, unlike Treg cells, CD4^+^CD25^−^Foxp3^+^ T cells also synthesize IFN-γ, IL-4, IL-2, and IL-17, although less than effector T cells [5]. Thus, we suggest that CD4^+^CD25^−^Foxp3^+^ T cells have both suppressive and proinflammatory functions in RA patients, which are between those of Treg cells and effector T cells. We also found that the proportion of CD4^+^CD25^+^Foxp3^+^ Treg cells from RA patients was significantly increased compared with that from HCs. However, the expression levels of CTLA-4 and GITR were both significantly decreased, and the synthesis of IL-17 was increased. Collectively, these data indicate that Tregs display a distinct phenotype, whereas the suppressive capacity of Treg cells in the PB of RA patients was defective. Treg cells may exhibit some proinflammatory features in RA.

Treg cells can suppress B-cell responses and B-cell-mediated antibody production [22]. Treg cell depletion can reduce plasma cell (PC) populations during systemic infection, and CTLA-4 deletion in Treg cells results in elevated PCs [23]. Our data showed that the proportion of CD4^+^CD25^−^Foxp3^+^ T cells was positively correlated with IgG titer and anti-CCP titer and negatively correlated with IgM titer. The proportion of CD4^+^CD25^+^Foxp3^+^ Treg cells was positively correlated with anti-CCP. This implied that the abnormal proportion and impaired suppressive function of CD4^+^CD25^−^Foxp3^+^ T cells and Treg cells in RA patients might be connected with autoantibody production.

In summary, we demonstrated an increased proportion of CD4^+^CD25^−^Foxp3^+^ T cells in untreated RA patients and was positively correlated with disease activity. Meanwhile, we suggest that CD4^+^CD25^−^Foxp3^+^ T cells in RA may serve as functional characteristic intermediates between Treg cells and effector T cells, which have both suppressive and proinflammatory features.

## Data Availability

The datasets supporting the conclusions of this article are included within the article.

## Abbreviations

PB: Peripheral blood
RA: Rheumatoid arthritis
CTLA4: Cytotoxic T-lymphocyte associated protein 4
GITR: Glucocorticoid-induced tumor necrosis factor receptor
ICOS: Inducible costimulatory
DAS28: Disease Activity Score in 28 joints
DAS28-ESR: The Disease Activity Score in 28 joints using the erythrocyte sedimentation rate
ESR: Erythrocyte sedimentation rate
CRP: C-reactive protein
RFs: Rheumatoid factors
anti-CCP: Anti-citrullinated protein antibodies
